# Neuroimmune cross-talk in *Leptospira*-associated acute encephalopathy syndrome

**DOI:** 10.3389/fimmu.2026.1831261

**Published:** 2026-06-04

**Authors:** Khushboo Sharma, Neha Tiwari, Nalini Mishra, Manish Sharma

**Affiliations:** 1 Department of Zoology, Banaras Hindu University, Varanasi, Uttar Pradesh, India; 2 Indian Council of Medical Research (ICMR) - Regional Medical Research Center (RMRC) Gorakhpur, Gorakhpur, Uttar Pradesh, India; 3 Molecular Parasitology Laboratory, Department of Zoology, University of Allahabad, Prayagraj, Uttar Pradesh, India

**Keywords:** acute encephalitis syndrome (AES), central nervous system (CNS), *Leptospira*, neuroinflamamation, peripheral immune response

## Abstract

The *Leptospira*-associated acute encephalopathy syndrome (AES) is a severe neurological complication, largely affecting the endemic regions. Unlike other classical neurotropic infections, *Leptospira*-induced encephalopathy is mainly induced via immune-mediated mechanisms through dysregulation of glial response and peripheral immunity. The onset of infection is marked by the invasion of early innate immune clearance. There is a substantial presence of bacteremia and elevated peripheral inflammation due to the atypical engagement of pattern recognition receptors. The enhanced circulating cytokines and endothelial dysfunction cause blood–brain barrier disruption, along with the activation of nuclear factor-kappa B (NF-κB) and mitogen-activated protein kinase (MAPK) cascades. The subpopulations of the glial cells are the primary central nervous system (CNS) populations that undergo activation, such as microglia and astrocytes, to re-establish homeostasis. There is a positive feedback loop activation for the inflammation pathway, with exacerbated cerebral edema and neuronal dysfunction, which are characteristic of AES. The severity of neuronal parasitic disease correlated with immune dysregulation and glial activation rather than the direct *Leptospira* infection in the neuronal tissue. It may be proposed that the *Leptospira*-induced AES represents a neuroimmune disorder in which peripheral immune activation and glial-driven neuroinflammation converge to produce acute cerebral dysfunction. Understanding these interconnected pathways is essential for improving diagnosis and developing targeted therapeutic strategies for Leptospirosis/*Leptospira*-associated AES.

## 1 Introduction

*Leptospira* spp. are the cause of leptospirosis, sometimes referred to as “rat-urine disease”, a neglected zoonotic and waterborne illness. Both direct and indirect contact with the urine and feces of sick animals can spread this illness (1). The disease is distributed worldwide and exhibits a wide clinical spectrum ranging from asymptomatic or mild febrile illness to a severe form characterized by icterus and multi-organ failure (2).

Globally, leptospirosis contributes significantly to morbidity and death; however, its true burden remains underestimated because of underreporting and diagnostic challenges. Nevertheless, the prognosis of disease is difficult and is often neglected, thus making it difficult to determine its actual scope and severity. The disease is strongly associated with environmental and socio-economic factors, including high rainfall, humidity, poor sanitation, and inadequate drainage systems that facilitate the perpetuation of bacterial survival and transmission. The workers involved in animal husbandry, agriculture, and sewage cleaning are at higher risk of contracting the disease (3).

Despite its public health importance, leptospirosis and its neurological complications remain underrecognized, particularly in endemic regions. The inability to recognize the clinical symptoms due to overlap with other febrile illnesses often results in delayed diagnosis and missed cases (4, 5). Inadequate clinical awareness and limited integration into surveillance systems further contribute to underestimation of disease burden, highlighting the need for improved diagnostic tools and integrated surveillance frameworks (6).

The World Health Organization (WHO) estimates that there is a higher dominance of leptospirosis in tropical and subtropical regions, largely affecting endemic regions such as Southeast Asia, portions of Africa, and Latin America (7–9). There are clinical similarities in leptospiral AES and other encephalitis forms of disease; the clear prognosis of disease is difficult to ascertain, resulting in the poor documentation of the disease. For example, in the African continent, the cases remain underreported due to constraints in the technological diagnostics, resulting in one of the lowest documented incidence rates globally (10). Although the disease ranks as the predominant contributor in the zoonotic disease category that causes morbidity and mortality (11), the correct data and documentation with respect to disease prevalence are unknown. Among Southeast Asian countries, leptospirosis is highly prevalent in coastal states of India and endemic to states such as Uttar Pradesh (7, 8, 12, 13). Hence, there is a clear need to enhance awareness among healthcare providers and policymakers regarding the human health burden of leptospirosis in tropical and subtropical regions of the world.

Leptospiral infection presents with diverse clinical manifestations, including Weil’s syndrome, characterized by jaundice and renal failure (14, 15). Physiologically, the disease may progress through an immune phase associated with elevated inflammatory mediators, contributing to enhanced severity of the disease (16). Among these complications, AES is one of the neurological consequences of leptospirosis that is becoming more broadly recognized, as per the recent epidemiological and clinical investigations. Neurological manifestations may result from direct invasion of the central nervous system (CNS) or immune-mediated mechanisms, commonly presenting as meningitis, followed by peripheral neuropathies (17).

### 1.1 Biology and pathogenesis of *Leptospira*

The taxonomy of *Leptospira* has undergone significant evolution over the past century. The isolation of the organism was reported in 1915 for the first time at Kyushu University Medical School in Fukuoka (18). Following that, the serological classification in 1954 established that the suspensions of living *Leptospira* were agglutinated when exposed to sera from patients suspected of suffering from Weil’s disease (19). In 1962, the genus was divided into *Leptospira interrogans* and *Leptospira biflexa*, and the term “serotype” was replaced by “serovar” in 1972. Between 1991 and 1998, 16S rRNA phylogenetic studies identified three major clades: pathogenic, saprophytic, and intermediate (20). Currently, the genus comprises multiple species with varying pathogenic potential. In India, isolation studies have identified *L. interrogans* as the predominant pathogenic species, while other isolates have been classified under *L. kirschneri* and *L. noguchii* (21).

Leptospirosis is a zoonotic disease that can be transmitted via exposure to the urine of infected animals, particularly rodents, cattle, pigs, and dogs, which serve as intermediate hosts (22). Humans get the infection most commonly through occupational exposure, flooding, and poor sanitation (18). According to the WHO, outbreaks are mostly associated with rainfall and flooding that facilitates the dissemination of the organism in surface water and increases human contact with contaminated sources. Transmission begins when the spirochetes enter the body through cuts, wounds, or mucous membranes of the eyes, nose, or mouth; ingestion of contaminated water may also contribute (18). Following entry, *Leptospira* rapidly penetrates into the host tissues owing to their motility and ability to traverse cell junctions, gaining access to the bloodstream and causing leptospiremia within hours to days (23). The bacteria disseminate hematogenously to multiple organs (such as the liver and CNS) during the septicemic phase (22). Adhesion to host extracellular matrix components and evasion of complement-mediated killing through surface proteins facilitate the systemic spread and persistence (24). The Centers for Disease Control and Prevention notes that environmental exposure through contaminated water is the most common route globally, particularly in endemic regions. Thus, transmission is closely linked to environmental contamination, occupational risk, and host exposure to compromised skin or mucosal barriers, while host entry is facilitated by bacterial motility, tissue penetration mechanisms, and early bloodstream dissemination (18).

### 1.2 Acute encephalitis syndrome and *Leptospira* spp.

**Figure 1:**
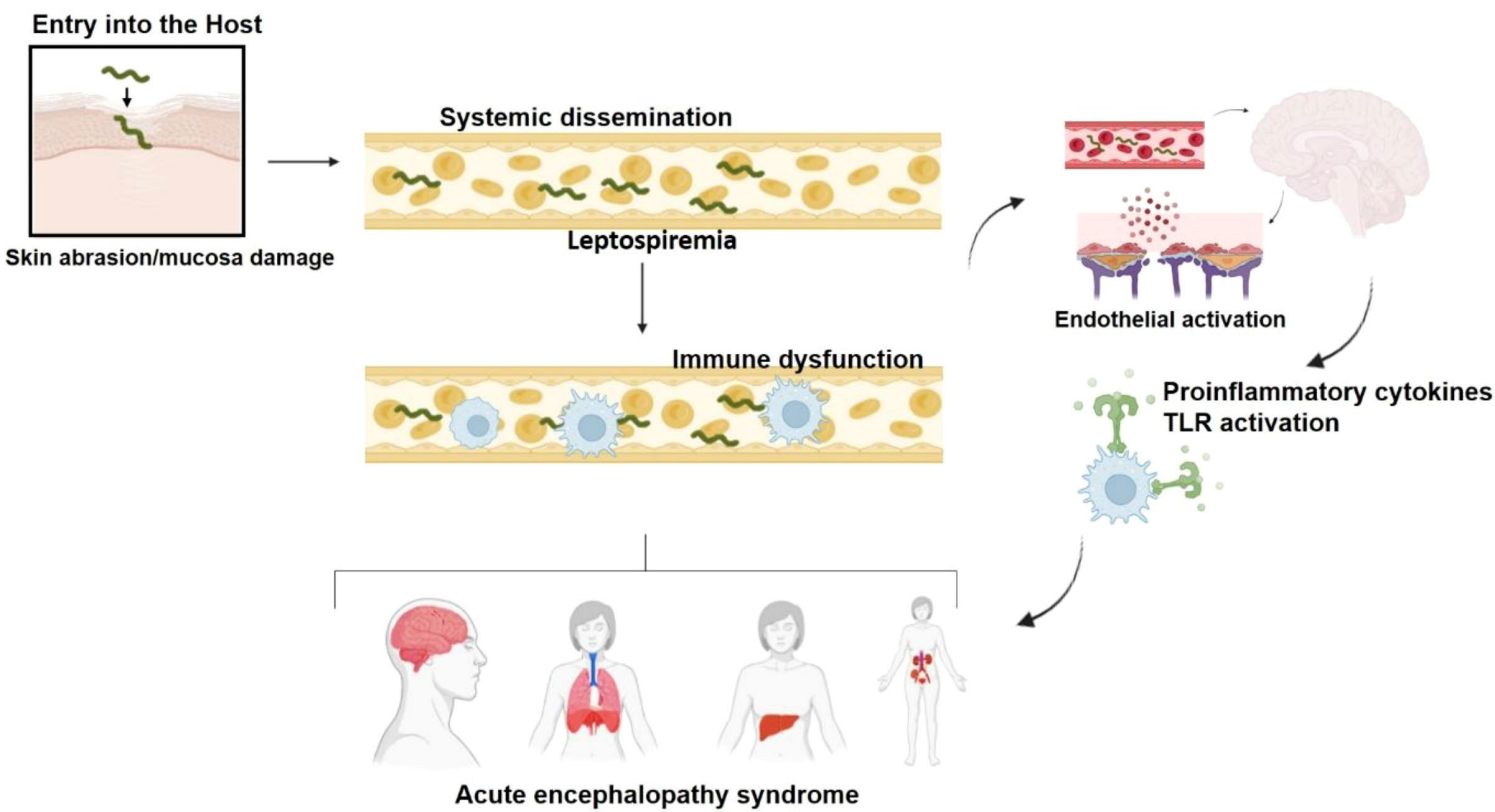
Systemic dissemination and immune activation during leptospiral infection. *Leptospira* enter through damaged skin or mucosa and disseminate via the bloodstream (leptospiremia). Circulating bacteria trigger innate immune activation and proinflammatory cytokine. They are released through TLR signaling, leading to endothelial activation and vascular inflammation. Systemic immune dysregulation promotes multi-organ involvement, including the lungs, liver, kidneys, and the central nervous system. In severe cases, neuroinflammation contributes to the development of AES (BioRender).

AES encompasses an acute febrile illness with neurological dysfunction, including altered consciousness, disorientation, delirium, or coma, attributable to either infectious or non-infectious causes (25). *Leptospira* species typically obtain access to various tissues or organs through the bloodstream after entering it through skin injury (**Figure 1**). The CNS may be affected either directly through leptospiral parenchymal invasion or indirectly as a consequence of hepatorenal dysfunction (26). Adopting encephalitis diagnostic frameworks that incorporate major and minor criteria strengthens the diagnosis of AES in clinical and research settings. While supportive (minor) criteria include fever ≥38 °C, focal neurological deficits, cerebrospinal fluid (CSF) pleocytosis (>5 cells/mm³), characteristic abnormalities on neuroimaging [computed tomography (CT)/magnetic resonance imaging (MRI)], or electroencephalographic (EEG) changes suggestive of encephalitic activity, major criteria include persistent altered mental status lasting ≥24 h with no other explanation (27). Diagnostic confidence is increased when the principal criterion is present along with two or more supporting characteristics (25). Although they are not required for the initial case definition, laboratory tests are essential in identifying the etiological cause of AES. Pathogen-specific IgM antibody detection in serum or CSF, polymerase chain reaction (PCR) for bacterial or viral nucleic acids, and, less frequently, antigen detection or culture are methods used for etiological confirmation. AES cases are categorized as laboratory-confirmed, AES caused by non-JE agents, or AES of unknown etiology, which is still prevalent in endemic areas, based on laboratory results (28).

The neuropathological hallmarks of AES in the CNS are the diffuse or focal neuroinflammation, followed by perivascular lymphocytic infiltration, microglial activation, and astrocytosis. These findings indicate an activated innate immune response within the CNS (27). In encephalitic disorders, cerebral edema, both cytotoxic and vasogenic, is commonly reported. Furthermore, they play a significant role in the elevation of intracranial pressure, altered consciousness, and poor clinical outcomes (29). Vascular pathology, including endothelial activation, capillary leakage, and occasional microhemorrhages, has been reported in AES, underscoring the role of vascular dysfunction in disease pathogenesis (30). Neuropathological alterations in infections such as neuroleptospirosis result mostly from immune-mediated processes and endothelium damage rather than widespread pathogen invasion. These alterations might occasionally take the form of demyelinating lesions that resemble acute disseminated encephalomyelitis (26). .

### 1.3 Distinction from viral encephalitis

Distinguishing leptospirosis AES from acute encephalitis is clinically important due to overlapping presentations but differing pathophysiological mechanisms. Leptospiral AES often presents as part of a systemic bacterial infection, with extra-neurological features such as jaundice, renal dysfunction, rash, and hepatic involvement that are less common in purely viral encephalitis and may reflect underlying leptospiral multisystem involvement (31).

Pathophysiologically, leptospiral CNS involvement is associated mainly with the immune-mediated vasculitis and inflammatory mechanisms due to circulating immune complexes and cytokine activation rather than extensive microbial invasion of the brain parenchyma, whereas viral encephalitis frequently results from direct viral replication within neurons and glial cells, causing neuronal death and inflammatory responses (32). CSF findings may also differ in the two, with viral encephalitis often associated with higher lymphocytic pleocytosis and characteristic viral PCR positivity, while leptospiral AES may show pleocytosis in the context of systemic inflammation and may require serological tests for confirmation (33).

### 1.4 Correlation of immune dysregulation with disease severity

The severity of leptospirosis, including neurological manifestations such as AES, is closely linked to immunological dysregulation. It has been reported that there is elevation in the levels of pro-inflammatory cytokines, especially during the immune phase of illness (22). For example, the increased levels of interleukin-6 (IL-6), interleukin-1β (IL-1β), and tumor necrosis factor-α (TNF-α) in the blood have been repeatedly linked to serious clinical symptoms (16), while the increased vascular permeability, disruption of the blood–brain barrier (BBB), and multiorgan dysfunction are all consequences of this cytokine-mediated inflammatory response, which also leads to endothelial activation and damage (30). An imbalance between pro-inflammatory and anti-inflammatory immune responses results in immune-mediated tissue damage rather than direct bacterial cytotoxicity (26).

Despite having the cumulative evidence that raises the issue of leptospirosis as a global health concern, its role in AES remains insufficiently characterized and addressed. The reports and existing literature mainly focus either on leptospirosis as a systemic infection or on AES as a heterogeneous clinical entity with multiple etiologies. However, a focused synthesis integrating leptospiral infection with AES, including epidemiology, pathogenesis, immune mechanisms, and diagnostic challenges, is lacking. Our review aims to address this gap by providing a comprehensive and integrated overview of *Leptospira*-associated AES, with particular emphasis on its neurological manifestations, underlying immunopathology, and challenges in clinical recognition and diagnosis. By consolidating dispersed evidence and highlighting underrecognized aspects of leptospiral neuro-involvement, this review explains the distinct perspective that bridges infectious disease biology with neurological outcomes, especially in endemic and resource-limited settings.

## 2 Innate immune recognition of *Leptospira*

**Figure 2:**
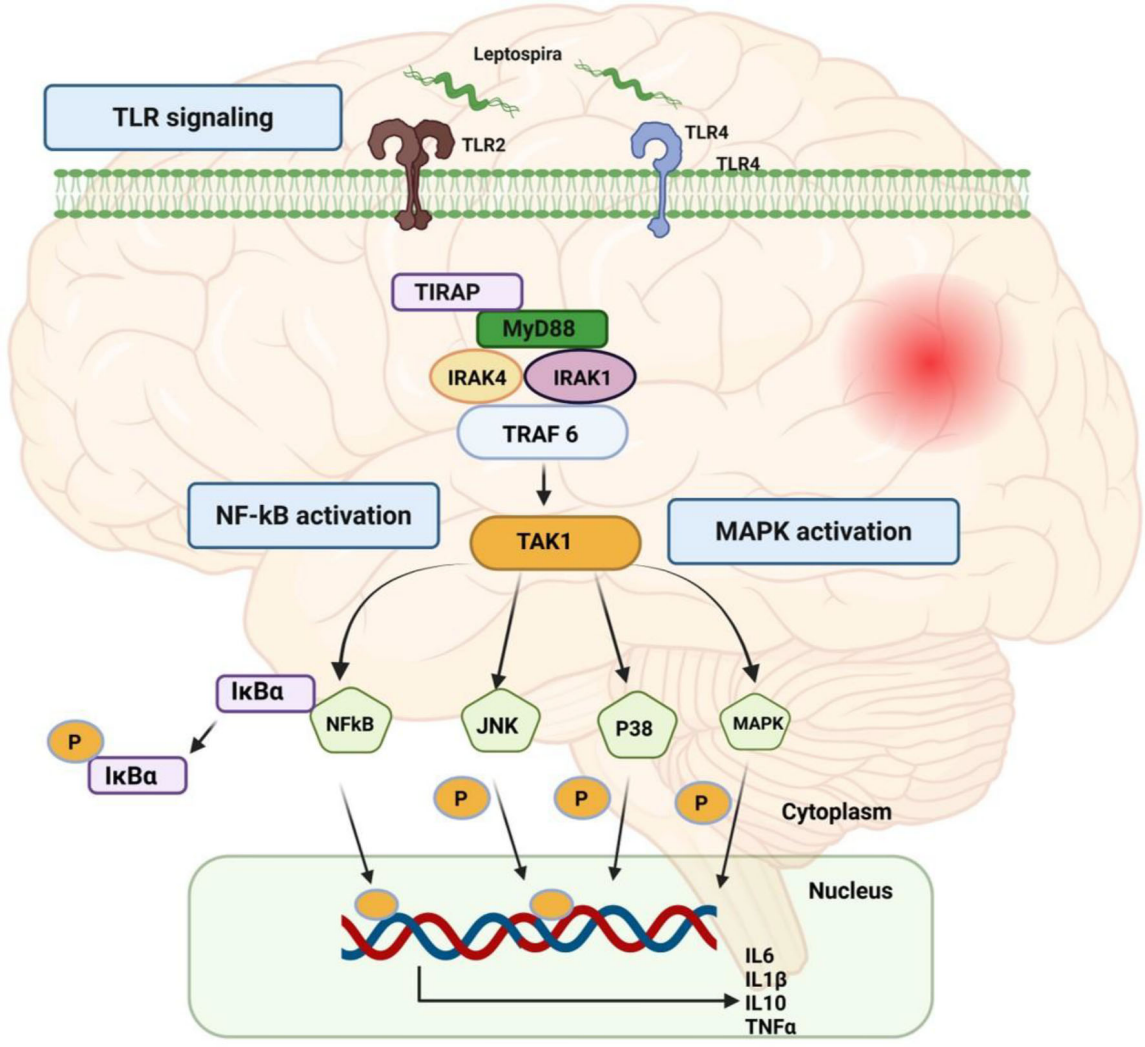
*Leptospira*-induced signaling cascade driving neuroinflammation and AES. Recognition of *Leptospira* components by pattern recognition receptors (TLR2 and TLR4) initiates innate immune signaling in host cells. Engagement of these receptors recruits adaptor proteins TIRAP and MyD88, leading to the activation of downstream kinases IRAK1/IRAK4 and the adaptor TRAF6. This signaling complex activates TAK1, which serves as a central node linking NF-κB and MAPK pathways. Phosphorylation and degradation of IκBα release NF-κB, enabling its nuclear translocation and transcriptional activation of inflammatory genes. Concurrently, MAPK signaling pathways including JNK, p38, and ERK/MAPK are activated, further amplifying inflammatory responses. These pathways collectively induce the production of pro-inflammatory cytokines such as IL-6, IL-1β, TNF-α, and IL-10, contributing to systemic inflammation and neuroimmune activation. The resulting cytokine surge and glial activation promote neuroinflammation and neuronal dysfunction, ultimately contributing to the development of AES during leptospiral infection (BioRender).

The innate immune response is triggered upon recognition of *Leptospira* by pattern recognition receptors (PRRs). These PRRs are expressed on macrophages, dendritic cells, neutrophils, and endothelial cells. Among PRRs, Toll-like receptors (TLRs) play a dominant role in sensing leptospiral pathogen-associated molecular patterns (PAMPs) (34). On the basis of pathogenicity, the leptospires are categorized into pathogenic, viz., *L. interrogans*, *L. borgpetersenii*, *L. weilii*, and *L. kirschneri*, and non-pathogenic, viz., *L. biflexa* species. The pathogenic spirochete invaders survive by escaping the natural defense mechanism of the host. Structurally, the *Leptospira* spp. have a typical cell wall composition. Unlike other spirochetes, lipopolysaccharides (LPS) are anchored to the outer membrane (35). Leptospiral LPS possesses a structurally atypical lipid A moiety with reduced endotoxic activity. In contrast to murine cells, leptospiral LPS activates TLR4 signaling; in human cells, it predominantly signals through TLR2 (35, 36). This species-specific recognition is attributed to differences in lipid A acylation and phosphate composition, which impair optimal interaction with human TLR4/MD-2 complexes (35). Such altered sensing may delay early immune clearance and contribute to dissemination. Leptospiral outer membrane lipoproteins such as LipL32, LipL21, and LipL41 are potent TLR2 agonists that activate macrophages via MyD88-dependent pathways (37). These lipoproteins induce early inflammatory responses via TNF-α, IL-6, and nitric oxide (**Figure 2**). Additionally, the bacterial peptidoglycan fragments are recognized by the cytosolic sensors such as NOD1 and NOD2, leading to RIP2-mediated nuclear factor-kappa B (NF-κB) activation (38). Recently, the NLRP3 inflammasome role in caspase-1 activation and IL-1β maturation in pathogenic leptospiral infection was reported (39). However, the inflammasome activation is strain-dependent and correlates with virulence. Dendritic cells also undergo activation upon leptospiral exposure, though pathogenic strains may impair their maturation and antigen-presenting capacity, potentially influencing downstream adaptive responses (40). It may be inferred that the PRR signaling initiates inflammatory transcriptional programs while simultaneously shaping adaptive immune priming.

## 3 Adaptive immune responses in leptospirosis

Adaptive cellular immunity plays a critical role in the control and resolution of leptospiral infection. A cytokine response could be a useful marker in understanding the pathogenesis of leptospirosis and thereby the outcome of the disease.

### 3.1 T-cell response and cytokines

It is known that the CD4^+^; T helper (Th) cells regulate the downstream immune responses through cytokine production and macrophage activation. During the initial phases of the leptospiral infection, there is activation of the pro-inflammatory response, a Th1-skewed response characterized by interferon-γ (IFN-γ) production, which results in the increased macrophage bactericidal activity and promotes removal of the pathogen (23). In the case of human leptospirosis, increased levels of pro-inflammatory cytokines such as IFN-γ, TNF-α, IL-6, and IL-8 have been detected in the blood during the acute phase of the disease, which indicates the involvement of Th1 responses in the host’s protective mechanisms against the infection (16, 41).

In the case of severe leptospirosis, it has been documented that there is simultaneous elevation of pro-inflammatory cytokines (such as IFN-γ, IL-6, and IL-8) and regulatory cytokines (such as IL-10), implying disequilibrium of the inflammatory response rather than polarized Th1 dominance alone (42). The enhanced cytokine production results in the pulmonary hemorrhagic syndrome, acute kidney injury, and increased mortality, consistent with immunopathologic mechanisms contributing to organ dysfunction (41).

It has been empirically evidenced that the T helper 17 (Th17)-associated pathway plays a crucial role in leptospirosis progression. There is an elevation of cytokines, which leads to the differentiation of T helper cells through IL-21, maintenance through IL-23, and effector functions through IL-17A (43). An elevation in the level of IL-17A leads to the activation of inflammatory pathways (44). Though the activation of the IL-17 response may have a role in the enhanced clearance of bacteria at the tissue interface, overactivation may lead to endothelial damage. Regulatory T cell-associated cytokines, especially IL-10, are constantly elevated in severe leptospirosis and most probably represent the compensatory anti-inflammatory response for limiting tissue injury (16, 41). However, persistent systemic inflammation in spite of increased IL-10 implies that regulatory mechanisms may be insufficient to restore immune homeostasis.

CD8^+^; cytotoxic T lymphocytes may also contribute to pathogen control through cytolytic activity and cytokine secretion, although their precise role in human leptospirosis remains incompletely defined. Experimental models and immunologic analyses demonstrate that leptospiral antigens can elicit cellular immune responses, but further studies are required to clarify the contribution of CD8^+^; T cells to protective immunity (23).

Taken together, leptospirosis is characterized by a complex cytokine milieu involving both pro-inflammatory and regulatory mediators that collectively influence disease severity, immune balance, and clinical outcome. While individual cytokines have been discussed in detail, their integrated roles in systemic inflammation and neurological complications such as acute encephalopathy syndrome (AES) are summarized below.

This integrated cytokine network highlights that disease progression in leptospirosis, including neurological complications such as AES, is driven by a finely balanced yet often dysregulated interaction between pro-inflammatory and regulatory mediators.

### 3.2 Humoral immunity

Protective immunity is largely mediated by antibodies targeting leptospiral LPS, which constitutes the dominant surface antigen and principal determinant of serovar specificity (45). Passive transfer experiments in hamsters have provided the empirical evidence that anti-LPS antibodies confer protection against homologous challenge, establishing the central role of LPS-directed humoral immunity (45).

When there is acute infection caused by LPS of *Leptospira*, immunoglobulin M (IgM) antibodies appear during the early phase of disease progression and might be utilized as a tool for the serological diagnostic assays such as the microscopic agglutination test/MAT (18, 46). It has been known that the IgM antibodies contribute to early opsonization and complement activation. This further facilitates the removal of bacteria via classical pathway activation and elevation in the process of phagocytosis. This is followed by the class-switching of the IgG antibodies, which further augments the opsonization and bactericidal activity using complement fixation and Fc receptor–mediated phagocytosis (22).

However, it is largely serovar-specific due to considerable structural heterogeneity in the O-antigen portion of *L. interrogans* LPS. There are over 250 pathogenic serovars known to date, and the large antigenic diversity of the O antigen portion is a major limiting factor (46, 47). This is a major problem that needs to be addressed for developing a universal vaccine against *Leptospira*, as whole-cell and bacterin vaccines elicit restricted anti-LPS antibodies that are specific to each serovar and not against conserved proteins (47).

### 3.3 Immune memory and serovar specificity

Protective immunity following natural leptospiral infection can be long-lasting but is largely restricted to the infecting (homologous) serovar. Reinfection with antigenically distinct serovars has been documented, particularly in endemic regions where multiple serovars co-circulate (18, 22). This limited cross-protection reflects the marked structural diversity of the O-antigen component of leptospiral LPS, which determines serovar specificity and serves as the dominant target of protective antibodies (46). Long-term humoral immunity is maintained by memory B cells and long-lived plasma cells that sustain circulating antibody titers against previously encountered serovars. Experimental studies in animal models demonstrate durable protection against homologous challenge but poor protection against heterologous serovars, further confirming serovar-restricted immunity (45). Although cellular memory responses may contribute to long-term immune surveillance, current evidence indicates that antibody-mediated mechanisms remain the principal correlate of protection in leptospirosis (48). The narrow serovar specificity of adaptive immune responses represents a major obstacle for universal vaccine development and underscores the need to target conserved protein antigens rather than variable LPS structures (46, 47). Severe leptospirosis represents a paradoxical state characterized by marked systemic inflammation coupled with impaired immune resolution.

## 4 Inflammatory signaling pathways driving systemic response

### 4.1 NF-κB and MAPK signaling: central drivers of inflammation

There is a crucial role of NF-κB and mitogen-activated protein kinase (MAPK) signaling cascades in *Leptospira*-induced inflammation. These pathways amplify cytokine production and regulate apoptosis, stress responses, and endothelial activation (49). Clinical studies in human leptospirosis have demonstrated that severe disease is associated with a broad elevation of pro- and anti-inflammatory cytokines relative to mild disease, a pattern consistent with a “cytokine storm” phenotype (41).

At the cellular level, following TLR engagement, adaptor proteins MyD88 and TIRAP recruit IRAK4 and IRAK1, which associate with TRAF6. This complex activates TAK1 (transforming growth factor-β-activated kinase 1), a pivotal signaling hub linking upstream receptor activation to both NF-κB and MAPK cascades (50). TAK1 activates the IKK complex, resulting in the phosphorylation and degradation of IκBα and the nuclear translocation of NF-κB (p65/p50). NF-κB induces the transcription of multiple pro-inflammatory mediators (summarized in **Table 1**) and inducible nitric oxide synthase (56). Simultaneously, TAK1 activates MAPKs—ERK, JNK, and p38—which regulate transcription factors such as AP-1 and CREB. Importantly, pathogenic *Leptospira* induce stronger and more sustained NF-κB and p38/JNK activation compared to saprophytic strains, indicating virulence-dependent modulation of host signaling (57).

**Table 1:** Key cytokines and chemokines in *Leptospira*-associated AES.

Category	Molecule	Source cells	Function	Role in AES pathogenesis	References
Pro-inflammatory cytokines	TNF-α	Macrophages, monocytes, microglia	Induces inflammation, endothelial activation	Promotes blood–brain barrier (BBB) disruption and vascular leakage	(41, 51)
	IL-1β	Macrophages, microglia	Fever, leukocyte recruitment	Enhances neuroinflammation and glial activation	(44, 52)
	IL-6	Macrophages, endothelial cells, astrocytes	Acute phase response	Correlates with disease severity and CNS involvement	(16, 41)
Anti-inflammatory cytokines	IL-10	T cells, macrophages	Suppresses inflammation	Indicates immune imbalance in severe disease	(41, 42)
Th1 cytokines	IFN-γ	CD4^+^; T cells, NK cells	Activates macrophages	Enhances pathogen clearance but contributes to inflammation	(23, 41)
Th17 cytokines	IL-17A	Th17 cells	Neutrophil recruitment	Contributes to endothelial damage and inflammation	(43, 44)
Chemokines (CC family)	CCL2 (MCP-1)	Macrophages, endothelial cells	Monocyte recruitment	Drives infiltration of monocytes into tissues	(44, 53)
	CCL3, CCL5	Immune cells	Leukocyte trafficking	Amplifies inflammatory cell recruitment	(44)
Chemokines (CXC family)	CXCL8 (IL-8)	Macrophages, endothelial cells	Neutrophil recruitment	Promotes neutrophil-mediated tissue injury	(41, 54)
Complement-related mediators	C3a, C5a	Complement system	Chemoattractant, inflammation	Enhances vascular permeability and cytokine release	(44, 55)

Sustained activation of NF-κB and MAPK pathways during *Leptospira* infection establishes a positive inflammatory feedback loop, whereby pro-inflammatory cytokines enhance their own production and amplify systemic immune signaling. Both TNF-α and IL-1β, products of NF-κB-driven transcription, act in autocrine and paracrine manners to further stimulate NF-κB activity, increase endothelial adhesion molecule expression, and promote recruitment of neutrophils and monocytes (41). .

### 4.2 Regulatory feedback mechanisms

NF-κB signaling is inherently self-limiting under physiological conditions through negative feedback regulators. For instance, IκBα interacts with NF-κB subunits in the cytoplasm, which prevents NF-κB subunits from entering the nucleus. A20, also known as TNFAIP3, is a negative feedback inhibitor of TLR pathways that deubiquitinates signaling molecules such as TRAF6 and RIP1 (58, 59). Likewise, the activity of MAPK pathways is negatively regulated by phosphatases such as MAPK phosphatase-1 (MKP-1/DUSP1), which dephosphorylates activated p38 and JNK to revert the signal to its basal state (60).

However, during the severe leptospiral infection, irresistible host–pathogen interactions and persistent PRR stimulation may supersede the regulatory circuits. The empirical documented data from sepsis and systemic inflammatory models validated that the sustained NF-κB and MAPK activation can persist despite negative feedback due to high cytokine loads and continual PRR engagement (58, 61). However, there are limited analogous studies in leptospirosis; the enhanced and prolonged cytokine profiles observed in severe human disease strongly implied the dysfunctional regulatory control of inflammatory signaling (41). Furthermore, leptospiral virulence factors may also interfere with negative feedback mechanisms. For instance, it has been reported that the bacterial pathogens alter ubiquitination pathways that regulate NF-κB termination, suggesting a potential mode by which persistent signaling might occur in leptospirosis as well (62).

Importantly, dysfunction or insufficient activity of these negative feedback regulators may contribute to prolonged NF-κB and MAPK activation, leading to sustained cytokine production. Such persistent inflammatory signaling is likely to exacerbate endothelial dysfunction and BBB disruption, thereby promoting downstream neuroinflammatory processes that contribute to the development of *Leptospira*-associated AES.

## 5 Peripheral immune responses and cellular mediators

### 5.1 Complement system interactions and serum resistance

Complement activation represents a critical early innate immune defense mechanism against *Leptospira* in the bloodstream, with the classical pathway initiated by antibody–antigen complexes and the lectin and alternative pathways enabling antibody-independent recognition of microbial surfaces; all three converge at C3 activation, leading to opsonization, generation of inflammatory fragments (C3a and C5a), and formation of the membrane attack complex (MAC). One of the characteristic features of the pathogenic *L. interrogans* is their resistance to complement-mediated killing in normal human serum, in contrast to saprophytic species such as *L. biflexa*, which are readily lysed (55). The pathogenic *Leptospira* regulates serum resistance by recruiting host complement regulatory proteins, including factor H (FH), C4b-binding protein (C4BP), and factor I, to their surface, where these regulators remain functionally active and inhibit C3 convertase formation, promote C3b inactivation, and prevent downstream MAC assembly (55, 63). Other surface-associated virulence factors such as the immunoglobulin-like proteins LigA and LigB of *L. interrogans* have been shown to bind FH and C4BP, thus regulating the alternative and classical pathways and increasing the survival of the bacteria in the presence of serum (64). This mechanism of evading the complement system allows for the maintenance of the bacteremia that characterizes the early stage of the disease process referred to as the leptospiremic phase. The complement activation fragments C3a and C5a have been shown to act as potent anaphylatoxins that attract leukocytes and increase cytokine production and vascular permeability and may thus play a role in the endothelial activation and vascular damage that occur in the disease process of leptospirosis (44).

### 5.2 Peripheral immune cell trafficking in leptospirosis

Peripheral immune cell movement during *Leptospira* infection features a large, often harmful, influx of inflammatory cells such as monocytes and neutrophils into affected organs. This process is caused by the widespread release of chemokines and cytokines into the body. The peripheral blood mononuclear macrophages are the dominant population of innate immunity that infiltrates the phagocytes in all the internal organs of *L. interrogans*-infected mice, which could account for the non-pyogenic infectious disease of leptospirosis.

**Figure 3:**
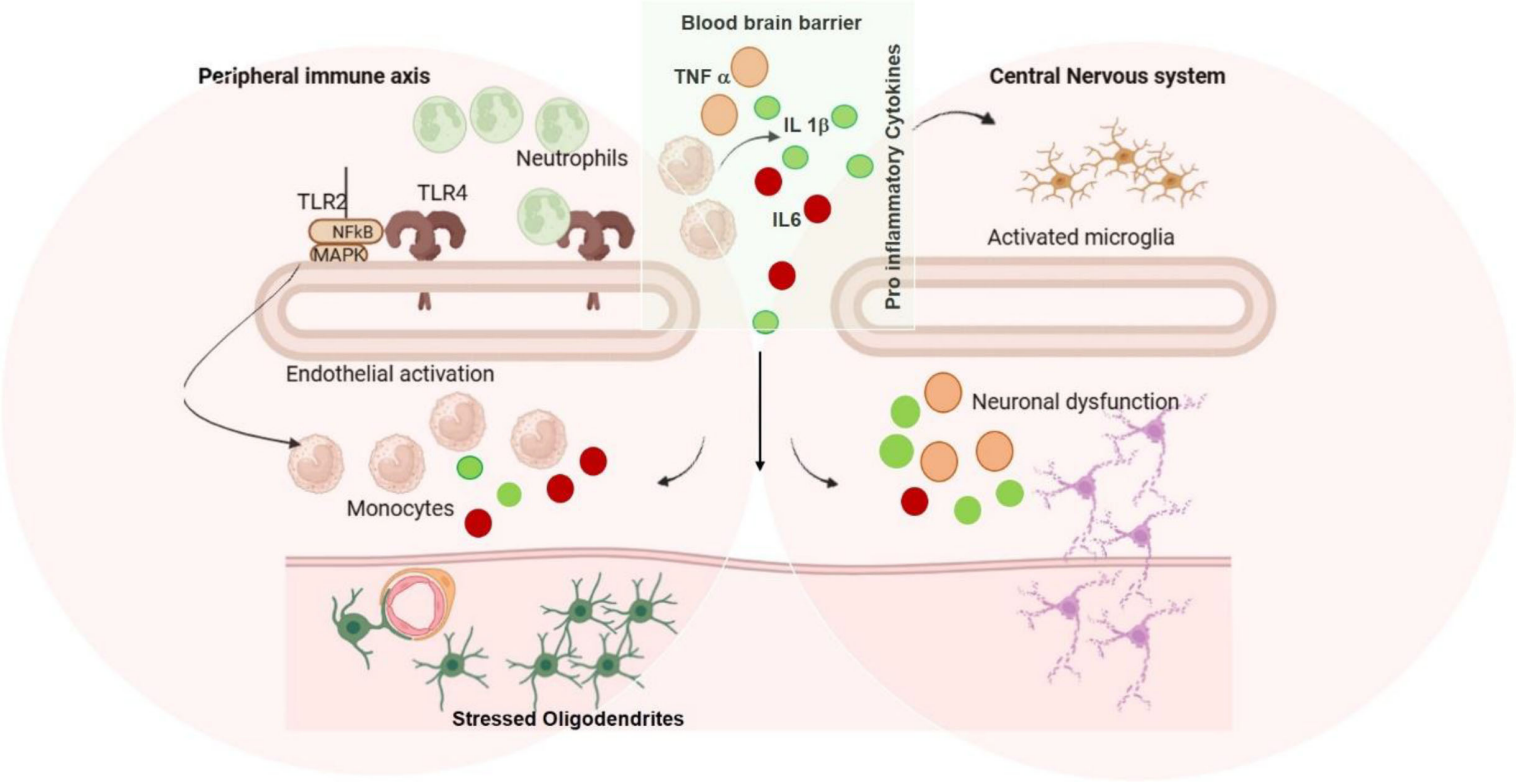
Peripheral–CNS immune cross-talk axis in *Leptospira*-associated neuroinflammation. Systemic leptospiral infection induces peripheral innate immune activation and robust production of proinflammatory cytokines (e.g., TNF-α, IL-1β, and IL-6) through TLR-mediated signaling. Circulating cytokines and activated immune cells promote endothelial activation and compromise blood–brain barrier integrity, enabling immune mediators and leukocytes to access the CNS. Within the brain, microglia and astrocytes respond to inflammatory signals, amplifying cytokine production and oxidative stress. This bidirectional communication between peripheral immunity and central glial cells sustains neuroinflammation, contributing to neuronal dysfunction and the development of AES (BioRender).

Chemokines and endothelial cell adhesion molecules directly mediate the migration of mononuclear macrophages and neutrophils from peripheral blood to sites of infection (**Figure 3**). The members in the CC family are chemokines of mononuclear macrophages, while the members in the CXC family are mostly chemotactic for neutrophils, where C stands for cysteine. There is elevation in the macrophage chemokines rather than neutrophil chemokines in the sera from patients with leptospirosis. It may be speculated that the elevation in the macrophage-specific chemokines and vascular endothelial cell adhesion molecules (VECAMs) may be the main mechanism responsible for peripheral blood mononuclear-macrophage infiltration during leptospirosis (53).

Neutrophil extracellular traps (NETs), which are made up of chromatin and associated with various antimicrobial proteins like histones, elastase, and myeloperoxidase, among others, act as a barrier that prevents the spread of pathogens. NETs trap and kill microbes extracellularly in a manner that is independent of phagocytosis and degranulation. NETosis is a critical mechanism of the host to prevent the spread of pathogenic *Leptospira* spp. NETs dramatically reduced *L. interrogans* survival but only for pathogenic species and not for saprophytes (54). Pathogenic leptospires have the ability to survive and disseminate to multiple organs after penetrating the host. The virulent *L. interrogans* can subvert the host immune system through the incorporation of complement regulatory factors such as complement FH and C4BP onto their surface. This interaction suppresses the activation of the complement system, thereby protecting the bacteria from lysis in the bloodstream and enhancing their survival in the host.

Pathological characteristics of leptospirosis are vasculitis and endothelial cell damage, leading to inflammatory infiltrates, localized ischemia, and hemorrhage in organs. *L*. *interrogans* possesses a collagenase, which is involved in the invasion and transmission of the pathogenic species (65). Endothelial permeability is controlled by the opening and closing of cell–cell junctions via the rearrangement of junction proteins and cytoskeletal proteins. The most prominent *L*. *interrogans* pathogenic phenotype was the disruption of adherens junctions. The pathogenic *Leptospira* strains target VE-cadherin/beta-catenin complexes, adherens junction proteins (66, 67).

Collectively, the results of these studies indicate that *L. interrogans* infection triggers a concerted response that involves the chemokine-dependent recruitment of monocytes and neutrophils and immune evasion through the modulation of the complement system, as well as the direct disruption of endothelial junctions to enhance systemic inflammation, vascular damage, and possibly organ and CNS involvement in leptospirosis. .

## 6 Neuronal cross-talk between peripheral immune dysregulation and the CNS axis

There is a direct effect of systemic inflammation on CNS functioning via neuronal, humoral, and cellular signaling cascades that are interconnected (52). The circulating inflammatory mediators such as pro-inflammatory cytokines and chemokines may bypass the specialized regions of the CNS where classical BBB are not involved and subsequently activate the resident glial cell populations of the CNS (68). The enhanced pro-inflammatory cytokines (summarized in **Table 1**) alter the BBB permeability and, as a consequence, activate the endothelial components of the BBB, which facilitates the peripheral immune response activation at the time of systemic infection (51). These mechanisms provide a structural and biological framework to understand the role of severe peripheral infections such as leptospirosis in contributing to neuroinflammatory complications such as encephalopathy.

The peripheral–CNS axis communication is driven by key mediators such as circulating cytokines and chemokines. There is an essential and direct role of the pro-inflammatory cytokines in neuronal health and physiological regulation via altering neurotransmitter metabolism, synaptic plasticity, and neuroendocrine signaling, which is well known (52). They are known to regulate immune trafficking in response to systemic infection (51, 69). The pro-inflammatory cytokines have been implicated in cognitive dysfunction during systemic inflammatory states, and chemokines enhance the leukocyte recruitment. Under severe systemic infections, they may activate the microglial and astrocytic responses, thus increasing the neuroinflammation even in the absence of direct microbial invasion.

There is a need to have a better understanding of CNS-immune surveillance. In the CNS meninges, the lymphatic vessels provide an alternative route for immune cell trafficking and antigen drainage from the CNS to peripheral lymph nodes (70). These meningeal lymphatics contribute to immune surveillance and may influence neuroinflammatory processes during systemic infection. The glymphatic system is a perivascular fluid transport system that relies on astrocytic aquaporin-4 channels to remove waste products, including inflammatory mediators, from the brain interstitium (71). Inflammation has been demonstrated to impair glymphatic flow, which might result in the accumulation of proinflammatory cytokines and potentially harmful substances in the CNS (72). The disruption of these waste removal pathways might contribute to increased levels of neuroinflammation during severe infectious conditions.

Peripheral CNS communication is bidirectional. While systemic cytokines can influence brain function, CNS-derived signals, including microglial cytokine production and activation of autonomic and hypothalamic–pituitary–adrenal (HPA) axis pathways, can modulate peripheral immune responses (52). Neural pathways, particularly vagal afferents, transmit inflammatory signals from the periphery to the brain, while efferent cholinergic pathways can suppress systemic cytokine production through the “inflammatory reflex” (73).

In severe systemic infections, dysregulation of this bidirectional axis may result in a feed-forward cycle of inflammation, endothelial dysfunction, and neuroimmune activation. Thus, CNS involvement in systemic inflammatory diseases likely reflects complex immune neural cross-talk rather than simple pathogen invasion.

### 6.1 Blood–brain barrier dysfunction during leptospirosis

The BBB plays a crucial role in keeping the immune system balanced within the CNS. Classically, the immune-privileged area, the CNS, depends on the BBB to regulate the entry of immune cells, cytokines, and pathogens from the body’s circulation. Structurally, the BBB endothelial cells are composed of tight junctions that control the paracellular transport and selectively regulate the movement of immune molecules. In addition to its role as a barrier, the BBB is crucial for immune signaling. The PRRs in the endothelial cells identify the inflammatory signals and start the initiation of the cascade for the release of the cytokines and chemokines. The glial cell population of the astrocytes and BBB component, pericytes, adds to this process by adjusting inflammatory responses and maintaining balance in neuroimmune activity. Under normal conditions, the BBB carefully controls leukocyte adhesion and migration. Nevertheless, at the time of the inflammation caused by the infection, the levels of adhesion molecules are elevated. For example, intercellular adhesion molecule-1 (ICAM-1) and vascular cell adhesion molecule-1 (VCAM-1) allow immune cells such as leukocytes and neutrophils to enter the CNS.

#### 6.1.1 Mechanisms of BBB disruption during leptospirosis

**Figure 4:**
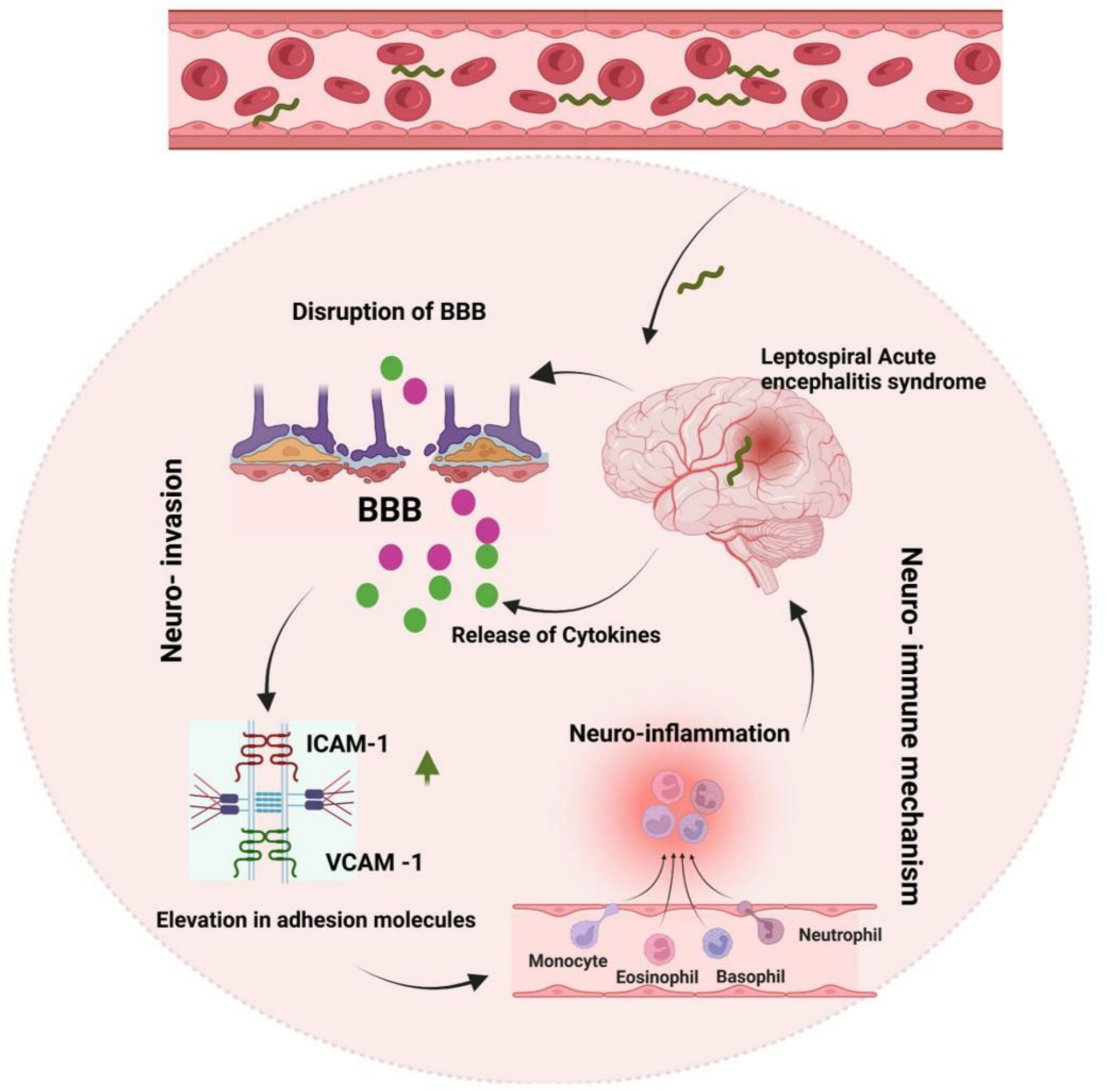
Blood–brain barrier disruption and neuroimmune mechanisms in *Leptospira*-associated AES. During systemic leptospiral infection, circulating bacteria and inflammatory mediators contribute to endothelial activation and disruption of the BBB. Increased production of pro-inflammatory cytokines promotes BBB permeability and facilitates neuroinvasion. Upregulation of endothelial adhesion molecules, including ICAM-1 and VCAM-1, enhances leukocyte adhesion and transmigration across the BBB. The infiltration of peripheral immune cells such as monocytes, neutrophils, eosinophils, and basophils further amplifies neuroinflammation within the central nervous system. This peripheral–central immune cross-talk leads to sustained inflammatory signaling, glial activation, and neuronal dysfunction, ultimately contributing to the development of *Leptospira*-associated AES (BioRender).

Endothelial cells are central targets of the inflammatory milieu during leptospiral infection (74). The activation of NF-kB in the endothelial cells elevated the expression of adhesion molecules involved in adherens junctions and tight junctions. The inflammation further facilitates the trans-endothelial migration of leukocytes via ICAM-1 and VCAM-1 (41, 75). Additionally, the increase in the expression of the circulating cytokines such as TNF-α acts as a positive feedback loop for endothelial activation due to bacterial infection. In leptospirosis, systemic inflammation and vascular leakage are widely observed. The systemic inflammation with the progression of disease results in acute infection in the respiratory and renal systems (76). These markers of vascular leakage and hemorrhage correlate with disease severity, implying that endothelial disruption is not a passive downstream consequence of cytokines but an active participant in organ dysfunction (**Figure 4**).

Concomitantly, the tight junction proteins are also reported to be involved in elevating the vascular permeability and systemic hemorrhage as a response to pro-inflammatory cytokines and chemokines during leptospirosis (77). Nevertheless, there are limited empirical studies to support the direct evidence supporting the role of tight junctions’ involvement. However, the systemic infection characterized by elevation in pro-inflammatory cytokines along with impaired endothelial integrity has been reported in individuals with neuroleptospirosis (44, 78). BBB impairment and elevation in cytokines in the form of a cytokine storm resulting in CNS inflammation have been observed as a response of systemic inflammation in many of the clinical and pathological studies (79). There is a direct effect of BBB integrity compromise on the CNS population of glial cells. The microglia and astrocytes further increase the level of neuroinflammation and result in the clinical manifestations of *Leptospira*-associated acute encephalopathy (44). .

### 6.2 Cellular landscape of neuroimmune interactions

While molecular signaling pathways such as NF-κB and MAPK activation provide important insights into the inflammatory cascade, a clear understanding of the cellular context is essential to define the core pathogenesis of *Leptospira*-associated AES. The neuroimmune response is mediated by coordinated interactions among distinct cellular populations across peripheral and central compartments, rather than by isolated molecular events (51, 52).

At the peripheral level, monocytes, macrophages, and neutrophils constitute the primary responders following leptospiral infection. These cells recognize leptospiral components through pattern recognition receptors and initiate robust inflammatory responses (35, 36). Monocytes and macrophages serve as major sources of pro-inflammatory cytokines such as TNF-α, IL-6, and IL-1β, thereby driving systemic inflammation and endothelial activation (16, 41). Neutrophils contribute through phagocytosis and the formation of NETs, which, while restricting pathogen dissemination, may also promote vascular injury and amplify inflammatory damage (44, 54).

Endothelial cells represent a critical cellular interface linking peripheral immune activation to CNS involvement. Upon exposure to circulating cytokines, endothelial cells undergo activation characterized by increased expression of adhesion molecules such as ICAM-1 and VCAM-1 (41, 75). This facilitates leukocyte adhesion and transendothelial migration, ultimately contributing to BBB disruption (51). Thus, endothelial cells function not only as structural barriers but also as active regulators of immune signaling and cellular trafficking into the CNS.

Within the CNS, microglia and astrocytes serve as the principal immune-responsive cell populations. Microglia, the resident macrophages of the brain, rapidly respond to systemic inflammatory cues by adopting activated phenotypes that release cytokines, chemokines, and reactive oxygen species (ROS) (80, 81). Astrocytes, in addition to maintaining BBB integrity, regulate neuroinflammatory responses through cytokine secretion and modulation of neuronal microenvironment (82, 83). Dysregulated activation of these glial cells amplifies neuroinflammation and contributes to cerebral edema and tissue dysfunction (51). Elevated cytokines and oxidative stress can impair synaptic transmission, disrupt neurotransmitter homeostasis, and lead to functional neuronal deficits even in the absence of direct bacterial invasion (52, 69). This supports the concept that neuronal dysfunction in *Leptospira*-associated AES is largely immune-mediated rather than a consequence of direct pathogen-induced cytotoxicity.

Collectively, these findings highlight that *Leptospira*-associated AES is driven by dynamic interactions among peripheral immune cells, endothelial cells, and CNS-resident glial populations. This cellular framework provides a clearer distinction from classical neurotropic infections and reinforces the concept of AES as a neuroimmune disorder arising from dysregulated cross-talk between systemic inflammation and brain-resident cells.

### 6.3 Innate immune-mediated inflammation in AES

While initial pathogen recognition through PRRs is essential for host defense, excessive activation of innate immune pathways contributes significantly to disease pathogenesis (35, 36). Systemic release of pro-inflammatory cytokines (summarized in **Table 1**) during the early phase of infection establishes a hyperinflammatory environment that affects both peripheral tissues and the CNS (16, 41, 52).

These circulating cytokines act on endothelial cells of the BBB, inducing the expression of adhesion molecules and increasing vascular permeability (51, 75). As a consequence, there is enhanced transmigration of innate immune cells, including monocytes and neutrophils, into the CNS. In parallel, activation of inflammasome pathways, particularly NLRP3, leads to further maturation and release of IL-1β, amplifying the inflammatory cascade (39, 44).

Within the CNS, innate immune activation is primarily mediated by microglia and astrocytes, which respond to peripheral inflammatory signals by producing additional cytokines, chemokines, and ROS (80–82). This creates a feed-forward loop of inflammation that exacerbates neuronal dysfunction. Importantly, this process occurs even in the absence of extensive bacterial invasion of the brain, supporting the concept that AES in leptospirosis is largely driven by immune-mediated mechanisms rather than direct pathogen-induced damage (26, 52).

Thus, dysregulated innate immune activation serves as a critical link between systemic infection and neuroinflammation, ultimately contributing to the clinical manifestations of AES, including altered consciousness, cerebral edema, and neurological impairment (29, 51).

### 6.4 Neuroendocrine regulation: role of the HPA axis in AES

The HPA axis represents a critical neuroendocrine pathway linking systemic inflammation to CNS responses during leptospiral infection (52, 84). Activation of the HPA axis is initiated by pro-inflammatory cytokines such as IL-1β, IL-6, and TNF-α, which stimulate the hypothalamic release of corticotropin-releasing hormone (CRH). This, in turn, induces secretion of adrenocorticotropic hormone (ACTH) from the pituitary gland, ultimately leading to glucocorticoid release from the adrenal cortex (84, 85).

Glucocorticoids exert potent immunomodulatory effects by suppressing excessive inflammatory responses and maintaining immune homeostasis (86, 87). Under physiological conditions, this negative feedback loop limits cytokine overproduction and prevents tissue damage. However, during severe systemic infections such as leptospirosis, persistent cytokine stimulation may dysregulate HPA axis function, resulting in either inadequate or excessive glucocorticoid responses (61, 88). Dysregulation of the HPA axis can contribute to the pathogenesis of AES through multiple mechanisms. Impaired glucocorticoid signaling may fail to control systemic inflammation, thereby exacerbating cytokine-mediated BBB disruption and neuroinflammation (51, 89). Conversely, prolonged glucocorticoid exposure may suppress protective immune responses and alter neuronal function, affecting cognition and consciousness (89).

Furthermore, bidirectional interactions between the HPA axis and CNS-resident immune cells, including microglia and astrocytes, influence neuroinflammatory signaling. Glucocorticoids can modulate microglial activation states, while inflammatory mediators from activated glial cells can further stimulate HPA axis activity, creating a feedback loop between endocrine and immune systems (52, 81).

Thus, the HPA axis serves as a crucial regulatory interface between systemic inflammation and CNS dysfunction in *Leptospira-*associated AES, and its dysregulation may significantly contribute to disease severity and neurological outcomes (52, 88).

## 7 Glial cell dysfunction in *Leptospira*-associated AES

Microglial activation in *Leptospira*-associated AES is primarily driven by systemic inflammation rather than direct bacterial invasion of the CNS. During severe leptospiral infection, elevated circulating cytokines such as TNF-α, IL-1β, and IL-6 disrupt BBB integrity and facilitate the entry of inflammatory mediators into the CNS (51, 52, 68). These peripheral signals act as key triggers for the activation of resident microglia, the principal innate immune cells of the brain.

Once activated, microglia play a central functional role in shaping the neuroinflammatory environment. They produce pro-inflammatory cytokines, chemokines, and ROS, which amplify local inflammation and contribute to neuronal dysfunction (80, 81, 90). In the context of AES, this exaggerated microglial response can disrupt synaptic signaling, alter neurotransmitter balance, and promote cerebral edema, ultimately contributing to altered consciousness and neurological impairment. Importantly, these processes can occur even in the absence of substantial leptospiral presence in the brain, reinforcing the concept that neuroinflammation is largely immune-mediated (52, 91).

Microglial activation has traditionally been categorized into pro-inflammatory (M1) and anti-inflammatory (M2) phenotypes. While this framework provides a simplified understanding of microglial function, it does not fully capture the complexity of microglial responses *in vivo*. Recent advances indicate that microglia exist along a spectrum of activation states that are highly context-dependent and influenced by the local inflammatory milieu, disease stage, and cellular interactions (92, 93). In *Leptospira*-associated AES, microglial responses are therefore better understood as dynamic and heterogeneous rather than restricted to discrete functional states.

**Figure 5:**
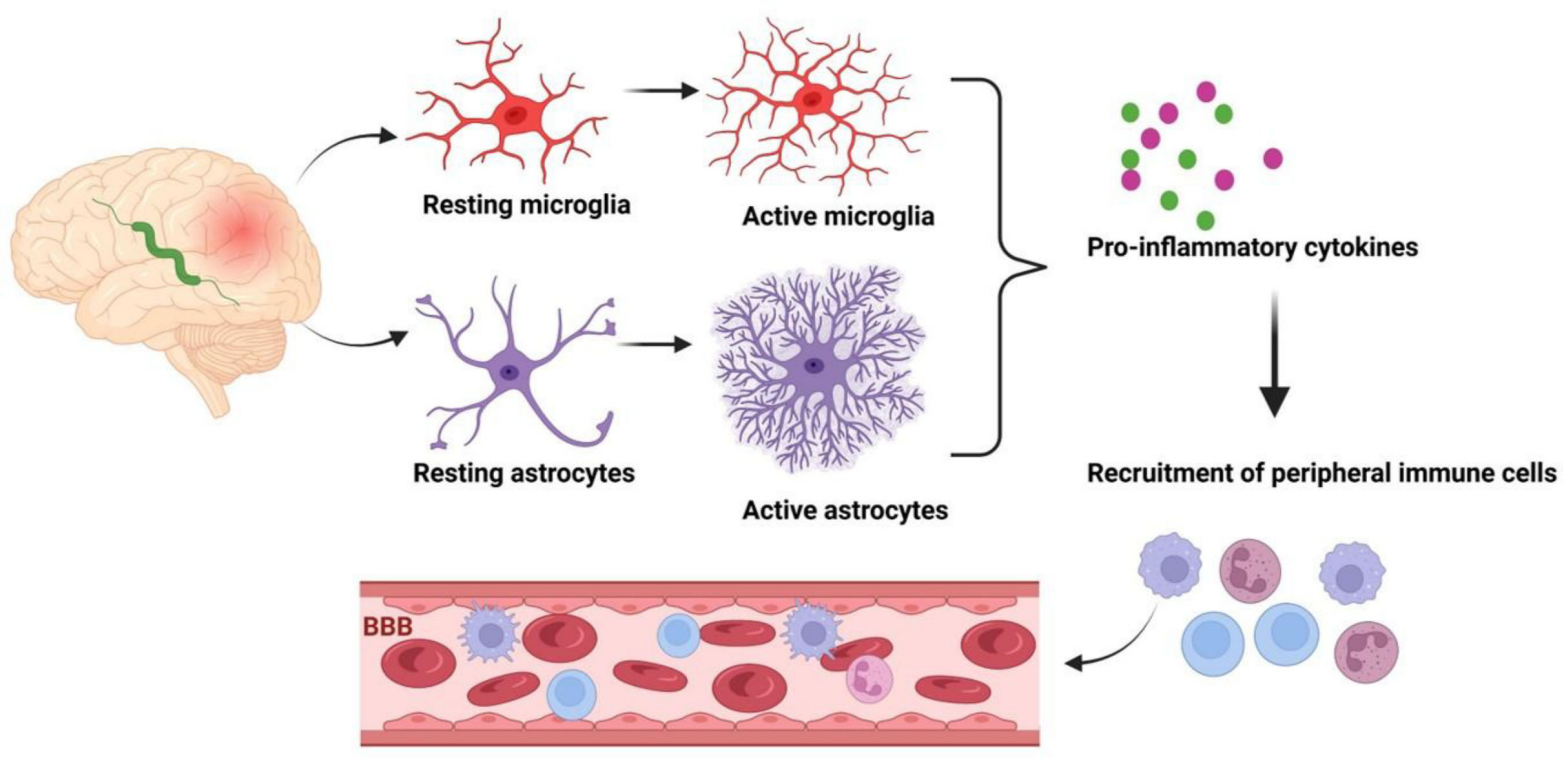
Glial activation and peripheral immune recruitment during *Leptospira*-induced neuroinflammation. Leptospiral infection in the central nervous system triggers activation of resident glial cells, including microglia and astrocytes. Resting microglia and astrocytes undergo morphological and functional changes upon sensing inflammatory stimuli, leading to their activated states. Activated glial cells release pro-inflammatory cytokines and chemokines, which amplify local inflammatory signaling. These mediators promote the recruitment and infiltration of peripheral immune cells across the BBB, including monocytes and other leukocytes. The interaction between activated glial cells and infiltrating immune cells enhances neuroinflammation and contributes to neuronal dysfunction associated with *Leptospira*-induced AES (BioRender).

Astrocytes also contribute significantly to neuroinflammation in AES. As key regulators of BBB integrity and neuronal homeostasis, astrocytes respond to inflammatory cues by undergoing reactive astrogliosis and producing cytokines and chemokines (82, 83). Disruption of astrocyte–endothelial interactions further compromise BBB stability, facilitating sustained immune cell infiltration and amplification of CNS inflammation (94). In parallel with microglial activation, astrocytic responses contribute to the persistence of the neuroinflammatory environment and exacerbate neuronal injury (**Figure 5**).

Collectively, glial cell dysfunction in *Leptospira*-associated AES reflects a coordinated response to systemic inflammation, involving both microglial activation and astrocyte reactivity. These processes form a self-amplifying inflammatory loop that links peripheral immune activation to CNS pathology, ultimately driving the neurological manifestations of AES.

## 8 Knowledge gaps and future directions

Despite major advancements in the understanding of the pathogenesis of leptospirosis, certain important aspects of the CNS infection have yet to be fully understood. While infection with *L. interrogans* has been linked to various manifestations of neuroinflammatory disease, including meningitis and encephalitis, the exact mechanisms of neuroinvasion of the bacteria and disruption of the BBB have yet to be fully understood. The exact molecular mechanisms of how the virulence factors of *Leptospira* induce alterations in the junctional proteins of the BBB, resulting in increased vascular permeability, have yet to be explored.

Another crucial gap in current understanding is related to the function of resident immune cells within the CNS, such as microglia and astrocytes, in the course of infection with *Leptospira*. While much is known about the systemic inflammatory responses and infiltration of immune cells in the periphery of infected individuals, little is known about their roles in contributing to neural damage and progression of disease in the context of glial cell activation. Furthermore, the relationship between *Leptospira* immune evasion mechanisms involving the complement system and neuroinflammatory responses within the CNS is not well understood.

Further research should concentrate on the identification of the factors that contribute to disruption of the BBB, the role of glial cells in the infection, and the mechanisms that govern the host–pathogen relationship, leading to neuroinflammation, and how this can aid in the identification of potential therapeutic targets in the prevention of neurological complications in leptospirosis.
